# PPARα induces cell apoptosis by destructing Bcl2

**DOI:** 10.18632/oncotarget.5988

**Published:** 2015-11-09

**Authors:** Jiaming Gao, Qian Liu, Ying Xu, Xin Gong, Runyun Zhang, Chenglin Zhou, Zhaoliang Su, Jianhua Jin, Haifeng Shi, Juanjuan Shi, Yongzhong Hou

**Affiliations:** ^1^ Department of Oncology, The Affiliated Wujin People's Hospital, Jiangsu University, Changzhou, Jiangsu Province, China; ^2^ Institute of Life Sciences, Jiangsu University, Zhenjiang, Jiangsu Province, China; ^3^ Jiangsu Taizhou People's Hospital, Jiangsu Province, China; ^4^ Department of Immunology & Laboratory Immunology, School of Medicine, Jiangsu University, Zhenjiang, Jiangsu Province, China

**Keywords:** ubiquitination, degradation, apoptosis, PPARα, Bcl2

## Abstract

PPARα belongs to the peroxisome-proliferator-activated receptors (PPARs) family, which plays a critical role in inhibiting cell proliferation and tumorigenesis, while the molecular mechanism is still unclear. Here we report that PPARα serves as an E3 ubiquitin ligase to govern Bcl2 protein stability. PPARα physically bound to Bcl2 protein. In this process, PPARα/C102 was critical for PPARα binding to BH3 domain of Bcl2, subsequently, PPARα transferred K48-linked polyubiquitin to lysine-22 site of Bcl2 resulting in its ubiquitination and proteasome-dependent degradation. Importantly, overexpression of PPARα enhanced cancer cell chemotherapy sensitivity. In contrast, silenced PPARα decreased this event. These findings revealed a novel mechanism of PPARα governed endogenous Bcl2 protein stability leading to reduced cancer cell chemoresistance, which provides a potential drug target for cancer treatment.

## INTRODUCTION

Apoptosis (programmed cell death) plays a critical role in maintenance of normal tissues homeostasis by elimination of the unwanted or damaged cells from organisms [1]. Evasion of apoptosis is a feature of many cancer cells that is involved in overexpression of Bcl2 (B-cell lymphoma 2) [2]. Bcl2 is a proto-oncogene to inhibit cell apoptosis in the tumor development. Deregulated expression of Bcl2 is linked to many human cancers, such as melanoma, breast, prostate, chronic lymphocytic leukemia, colon, and lung cancer [2–5]. The Bcl2 family proteins contain pro-survival proteins (Bcl2, Bcl-xl, Mcl1) and pro-apoptotic proteins (Bax, bad, Bim) [2, 3]. Under normal condition, Bcl2 constrains the pro-apoptotic proteins (Bax, Bak) to maintain the mitochondrial integrity and cell survival. In contrast, cytotoxic stimuli (chemotherapy or radiotherapy) activate pro-apoptotic proteins and induce cell apoptosis [1]. Although deregulated Bcl2 expression leads to impaired apoptosis that is a critical step in tumorigenesis, it is still unclear the mechanism to govern Bcl2 protein stability.

PPARα belongs to the peroxisome-proliferator-activated receptors (PPARs) family that contains PPARα, PPARδ, and PAPRγ [6–8]. PPARα plays a critical role in inhibiting cell proliferation and tumorigenesis. As a ligand-activated transcription factor, PPARα can be activated by fatty acids, fatty-acid derivatives LTB4 (leukotriene B4) and synthetic ligands [8, 9]. PPARα is the first identified PPARs that is expressed in skeletal muscle, liver, intestine, kidney, heart [10, 11], which inhibits tumorigenesis in different tissues, including to colon, breast, lung, lymphocytic leukemia, live, and ovarian cancer [12–18]. PPARα agonist fenofibrate induces mantle cell lymphoma apoptosis by activation of caspase-3 pathway [19]. Consistent with this, fenofibrate effectively induces primary glial tumor cell apoptosis by promoting FoxO3A phosphorylation [20]. Furthermore, clofibrate promotes hepatocarcinoma HepG2 cell apoptosis by increasing PPARα expression [21]. However, the direct molecular mechanism of PPARα-induced cell apoptosis is still unclear. Here we found that PPARα serves an E3 ubiquitin ligase to induce Bcl2 ubiquitination and degradation leading to cell apoptosis in response to chemotheraphy drugs.

## RESULTS

### PPARα induces Bcl2 degradation

**Figure 1:**
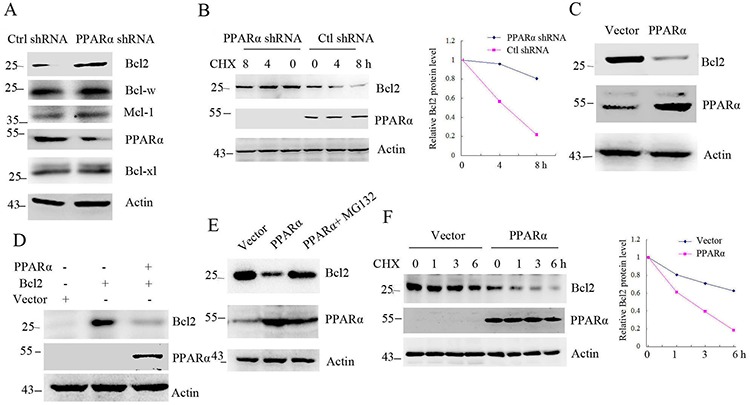
PPARα induces Bcl2 degradation **A.** Western blot analysis of SW480 cells transfected with control or PPARα shRNA. **B.** Bcl2 protein half-life was assayed by using CHX (30 μg/ml) in SW480 cells transfected with control or PPARα shRNA. The relative remaining Bcl2 protein levels following CHX treatment at each time point was calculated accordingly. **C, D.** HEK293T cells were transfected with plasmids as indicated. Cell lysates were subjected to Western blot. **E.** HeLa cells were transfected with vector, PPARα for 36 h. Cells were treated with or without 10 μM MG132 for 6 h before cell lysis. Cell lysates were subjected to Western blot. **F.** Bcl2 protein half-life was assayed by using CHX (30 μg/ml) in HEK293T cells transfected with vector or PPARα plasmid. The relative remaining Bcl2 protein levels following CHX treatment at each time point was calculated accordingly.

As a nuclear receptor protein, PPARα is expressed in cytoplasm and nucleus (Supplementary Figure S1). To detect the interaction of PPARα with Bcl2, SW480 cells were transfected PPARα shRNA. Western blot shows that silenced PPARα increased Bcl2 protein levels without effect on Mcl-1, Bcl-xl and Bcl-w pro-survival protein levels (Figure 1A). Consistent with this, Bcl2 protein half-life was significantly increased in PPARα silenced SW480 cells (Figure 1B). Overexpression of PPARα in HEK293T cells significantly decreased endogenous or exogenous Bcl2 protein levels (Figure 1C, 1D), but another two peroxisome-proliferator-activated receptors (PPARs) family members, PPARγ or PPARδ, had no effect on Bcl2 protein levels (Supplementary Figure S2). We next detected whether PPARα-reduced Bcl2 protein levels was involved in Bcl2 transcriptional activity. The analysis of RT-PCR and real-time PCR shows that PPARα had no effect on Bcl2 gene expression (Supplementary Figure S3A, S3B). To further detect whether reduced Bcl2 by PPARα was involved in proteasome-dependent degradation, overexpressed PPARα in HeLa cells were treated with MG132 (proteasome inhibitor). The results show that PPARα did not reduce Bcl2 protein levels in MG132 treatment cells (Figure 1E), suggesting that PPARα induced Bcl2 proteasome-dependent degradation. PPARα-induced Bcl2 degradation was demonstrated by half-life analysis (Figure 1F). We further detected whether the ligands of PPARα could effectively increase Bcl2 degradation. SW480 cells treated with fenofibrate, clofibrate or Wy-14,643 had no significantly effect on Bcl2 protein levels (Supplementary Figure S4). These findings suggest that PPARα induced Bcl2 proteasome-dependent degradation.

### PPARα serves as E3 ligase to induce Bcl2 ubiquitination

**Figure 2:**
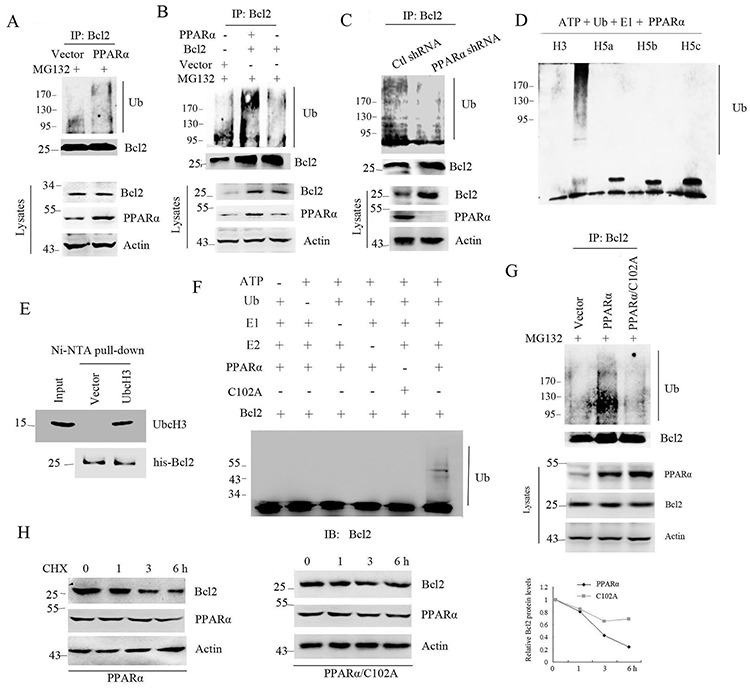
PPARα is an E3 ligase to induce Bcl2 ubiquitination **A, B.** HEK293T cells were transfected plasmids as indicated for 36 h. Cell lysates were subjected to denatured immunoprecipitation and Western blot. Cells were treated with MG132 (10 μM) for 6 h before cell lysis. **C.** SW480 cells were transfected with control or PPARα shRNA. Cell lysates were subjected to denatured immunoprecipitation and Western blot. **D.** *In vitro* polyubiquitin formation analysis was performed in reaction buffer contained different E2 (UbcH3, UbcH5a, b, c) with PPARα as indicated. Reactions were incubated at 30°C for 2 h. The ubiquitinated products were detected with ubiquitin antibody. **E.** Ni-NTA pull-down assay was performed to detect the interaction of PPARα with recombinant UbcH3. **F.** *In vitro* Bcl2 ubiquitination analysis was performed in the reaction buffer contained UbcH3, Bcl2 (10 μg) and 10 ng PPARα (WT or C102A) as indicated. Reactions were incubated at 30°C for 2 h. The ubiquitinated products were detected with Bcl2 antibody. **G.** HEK293T cells were transfected with vector, PPARα, or PPARα/C102A plasmids as indicated. Cell lysates were subjected to denatured immunoprecipitation and Western blot. Cells were treated with MG132 (10 μM) for 6 h before cell lysis. **H.** Bcl2 protein half-life was assayed by using CHX (30 μg/ml) in HEK293T cells transfected with PPARα or PPARα/C102A plasmids. The relative remaining Bcl2 protein levels following CHX treatment at each time point was calculated accordingly.

The targeted protein by proteasome undergoes ubiquitination and proteasome-dependent degradation [6, 22]. Although PPARα induced Bcl2 degradation, it is still unclear that whether PPARα could induce Bcl2 ubiquitination. Immunoprecipitation analysis shows that PPARα significantly induced endogenous and exogenous Bcl2 ubiquitination (Figure 2A, 2B). In contrast, silenced PPARα led to inhibition of Bcl2 ubiquitination (Figure 2C). As Bcl2 contains two zinc finger domains, the alignment analysis was performed by using several E3 ubiquitin ligases with ring domain. The results show that the first zinc finger domain developed a loop that is a potential E3 ligase (Supplementary Figure S5). To further identify whether PPARα has E3 ligase activity, *in vitro* ubiquitination analysis was performed. Our results show that UbcH3 but not UbcH5a/b/c was critical for PPARα-induced polyubiquitin formation (Figure 2D). Importantly, *in vitro* binding analysis shows that PPARα directly bound to UbcH3 (Figure 2E). LC/MS/MS analysis shows that PPARα induced K48-linked polyubiquitin formation (Supplementary Figure S6). To further detect whether PPARα was the E3 ligase for Bcl2, *in vitro* ubiquitination analysis was performed. The results show that PPARα significantly induced Bcl2 ubiquitination, but the PPARα/C102A mutant, the enzymic activity site, did not induce Bcl2 ubiquitination (Figure 2F). Consistent with this, PPARα/C102A had no effect on Bcl2 ubiquitination (Figure 2G) and protein half-life (Figure 2H). These findings suggest PPARα functions as an E3 ubiquitin ligase to induce Bcl2 ubiquitination and degradation.

### PPARα interacts with Bcl2

**Figure 3:**
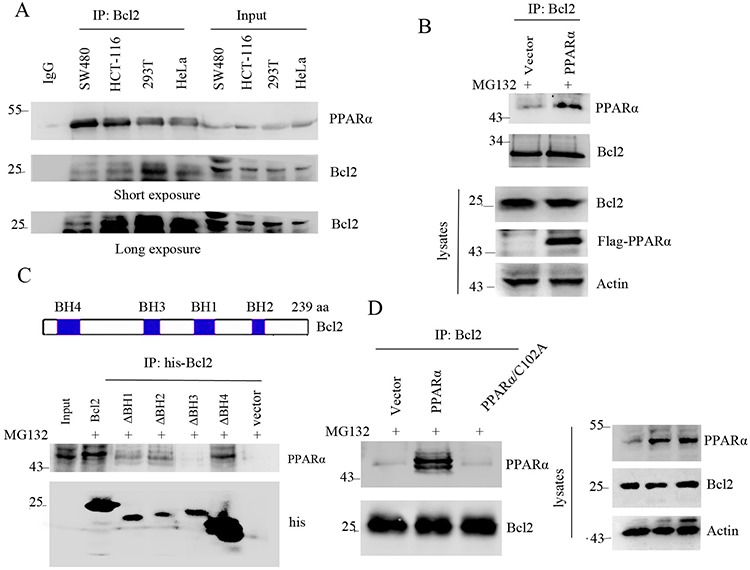
PPARα interacts with Bcl2 **A.** Cell lysates were subjected to immunoprecipitation and Western blot. **B.** HEK293T cells were transfected with vector or PPARα plasmids. Cell lysates were subjected to immunoprecipitation and Western blot. **C.** up panel shows the construct of Bcl2 protein. HEK293T cells were transfected with vector, Bcl2 wild type (WT) or mutant plasmids as indicated. Cell lysates were subjected to immunoprecipitation and Western blot. Cells were treated with MG132 (10 μM) for 6 h before cell lysis. **D.** HEK293T cells were transfected with vector, PPARα, PPARα/C102A mutant for 36 h. Cell lysates were subjected to immunoprecipitation and Western blot. Cells were treated with MG132 (10 μM) for 6 h before cell lysis.

Although the results have demonstrated that PPARα induced Bcl2 protein degradation, it is still unclear the direct interaction of PPARα with Bcl2. Immunoprecipitation analysis shows that PPARα physically bound to Bcl2 (Figure 3A). This was also consistent with overexpression of PPARα binding to Bcl2 (Figure 3B). Bcl2 protein contains BH1, BH2, BH3, and BH4 domains [3]. To detect the specific binding domain of PPARα to Bcl2, immunoprecipitation analysis shows that PPARα did not bind to deleted BH3 domain of Bcl2, suggesting that PPARα bound to BH3 domain of Bcl2 (Figure 3C). As C102 of PPARα was the critical enzymic site for PPARα-induced Bcl2 ubiquitination, further analysis shows that C102A mutant did not bind to Bcl2 (Figure 3D), suggesting that PPARα/C102 was critical for binding to Bcl2. These findings suggest that the physical interaction of Bcl2 with PPARα led to Bcl2 ubiquitination and degradation.

### Lysine-22 of Bcl2 is required for PPARα-induced it ubiquitination and degradation

**Figure 4:**
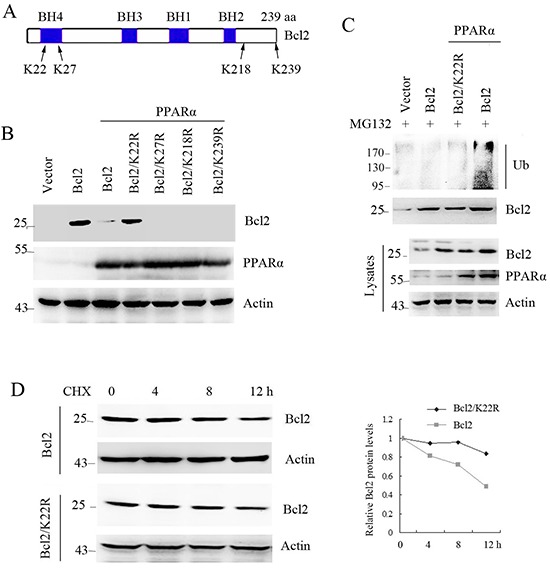
Lysine-22 of Bcl2 is required for PPARα-induced it ubiquitination and degradation **A.** construct of Bcl2 and the lysine sites. **B.** HEK293T cells were transfected with plasmids as indicated. Cell lysates were subjected to denatured immunoprecipitation and Western blot. Cells were treated with MG132 (10 μM) for 6 h before cell lysis. **C.** HEK293T cells were transfected with plasmids as indicated. Cell lysates were subjected to Western blot. **D.** Bcl2 protein half-life was assayed by using CHX (30 μg/ml) in SW480 cells transfected with Bcl2 or Bcl2/K22R plasmids. The relative remaining Bcl2 protein levels following CHX treatment at each time point was calculated accordingly.

Ubiquitin is attached to the lysine residue on a substrate that will be recognized and degraded by proteasome pathway [6, 22]. To identify the specific lysine site for binding to ubiquitin, the four lysine sites were replaced with arginine (Figure 4A). Immunoprecipitation analysis shows that PPARα did not induce Bcl2/K22R mutant ubiquitination (Figure 4B) and degradation (Figure 4C). Consistent with this, PPARα did not reduce Bcl2/K22R protein half-life (Figure 4D). These findings show that lysine-22 was required for PPARα-induced Bcl2 ubiquitination and degradation.

### PPARα/Bcl2 signaling increases cancer cell sensitivity in response to chemotherapy drugs

**Figure 5:**
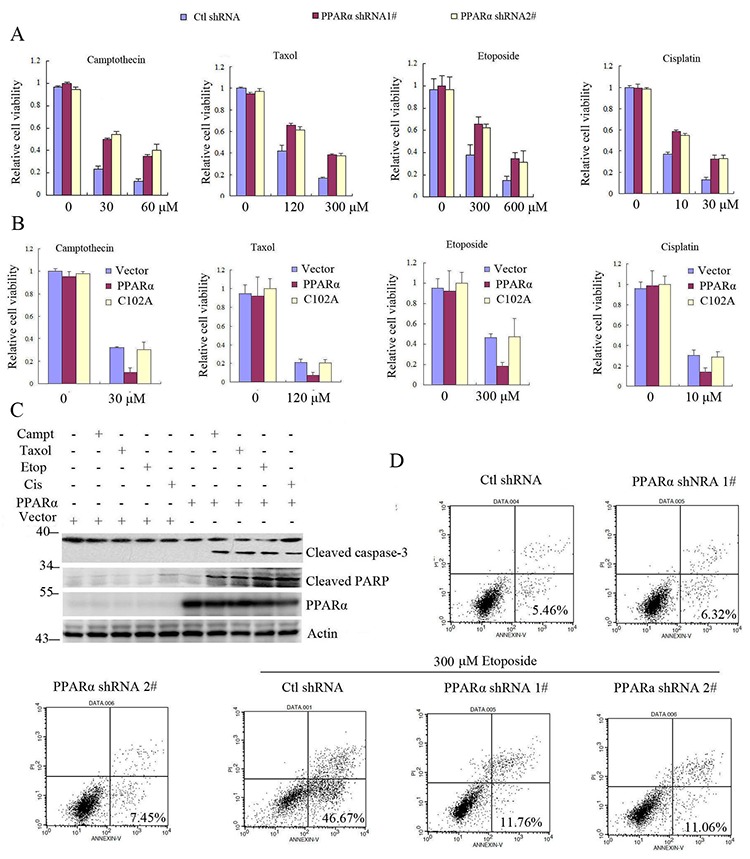
PPARα/Bcl2 signaling promotes cell apoptosis in response to chemotherapy drugs **A.** PPARα shRNA silenced SW480 cells were treated with chemotherapy drugs (camptothecin, taxol, etoposide, cisplatinum) as indicated for 24 h. Cell viability was assayed (see experimental procedures). Results are expressed as means ± SEM (*n* = 3). **B.** SW480 cells were transiently transfected with vector, PPARα, C102A plasmids as indicated. Cells were treated with chemotherapy drugs (camptothecin, taxol, etoposide, cisplatinum) for 24 h. Cell viability was assayed (see experimental procedures). Results are expressed as means ± SEM (*n* = 3). **C.** SW480 cells were transiently transfected plasmids as indicated. Cells were treated with camptothecin (60 μM), taxol (300 μM), etoposide (600 μM), and cisplatinum (30 μM) for 6 h. Cell lysates were subjected to Western blot. **D.** SW480 cells were transfected with control or PPARα shRNA as indicated. Cells were treated without or with etoposide for 24 h. Cell apoptosis was assayed (see experimental procedures).

Bcl2 promotes cell survival and inhibits apoptosis [2]. Our above data have demonstrated that PPARα induced Bcl2 ubiquitination and degradation. We further detected whether PPARα inhibited Bcl2-mediated cell survival. The results show that silence of PPARα did not affect cell viability, but significantly increased cancer cell viability in response to cytotoxic stimulation (camptothecin, toxal, cisplatin, etoposide) (Figure 5A, Supplementary Figure S7A, S7B). As C102 of PPARα was critical for Bcl2 ubiquitination and degradation, therefore PPARα not C102A significantly decreased cell viability in response to cytotoxic stimulation (Figure 5B, Supplementary Figure S8A). Similarly, PPARα did not reduce Bcl2/K22R mutant cell viability in response to cytotoxic stimulation (Supplementary Figure S9). As an anti-apoptotic protein, Bcl2 degradation by PPARα activated the downstream apoptotic signaling in response to chemotherapeutic agents (caspase-3, PARP-1) (Figure 5C) as well as increased apoptosis (Figure 5D, Supplementary Figure S8B). These findings suggest that PPARα induced Bcl2 ubiquitination and degradation leading to increased cancer cell chemotherapy sensitivity.

## DISCUSSION

Evasion of apoptosis is a feature of many cancer cells that is involved in overexpression of Bcl2, which inhibits cell apoptosis and enhances chemoresistance [2]. Aberrant expression of Bcl2 is associated with many human cancers, such as melanoma, breast, prostate, chronic lymphocytic leukemia, colon, and lung cancer [2–5]. Therefore, inhibition of Bcl2 expression contributes to cancer therapy. Here we show that PPARα serves as a novel E3 ubiquitin ligase to induce Bcl2 ubiquitination and degradation. The canonical E3 ubiquitin ligases contain RING finger domain, which induces the substrate proteins for ubiquitination and degradation [23]. PPARα contains two zinc finger domains, and the cooperation of two zinc finger domains developed a RING domain. The PPARα E3 ubiquitin ligase activity was identified by *in vitro* ubiquitination analysis. The results show that UbcH3 but not UbcH5a/b/c was critical for PPARα-induced polyubiquitination formation, suggesting that PPARα was a novel E3 ubiquitin ligase. RING domains possess conserved Cys and His residues, which are critical for ubiquitin ligase activity [23]. As the residues of Y (number 3) and V (number 4) are not conserved C or H in loop 2 of PPARα, suggesting that loop1 could be a critical domain for PPARα E3 ligase activity. Consistent with this, C102A mutant of PPARα inhibited its E3 ligase activity. Previous report shows that PPARα enhances the colon cancer cell sensitivity in response to hydroxycamptothecin (HCPT) [24], while the mechanism is still unclear. Further analysis shows that PPARα significantly increased cancer cell sensitivity in response to chemotherapy drugs. These findings were consistent with PPARα-induced Bcl2 degradation. PPARα agonists (fenofibrate, Wyeth14,643, clofibrate) can induce cancer cell apoptosis and inhibit tumor angiogenesis in a PPARα dependent or independent manner [19, 21, 25–27], while our results show that these ligands had no significant effect on Bcl2 protein levels.

Taken together, PPARα functions as an E3 ubiquitin ligase to induce Bcl2 ubiquitination and degradation, leading to increased cancer cell sensitivity in response to chemotherapy drugs. These findings provide a potential drug target for cancer treatment.

## MATERIALS AND METHODS

### Cell culture, treatment and reagents

The human SW480, HCT-116, HEK293T, HeLa cells were obtained from the ATCC. These cells were cultured in DMEM supplemented with 10% fetal bovine serum (FBS). For protein half-life analysis, cells were treated with cycloheximide (CHX, 30 μg/ml, Sigma). Protease inhibitor cocktail (Sigma). Fenofibrate, wy-14,643 and clofibrate were purchased from Toronto Research Chemical Inc. Geneticin (G418 sulfate) was purchased from Life Technologies. Taxol (Ruibio), cisplatinum (Tokoyo Chemical industry, Japan). Etoposide and camptothecin (Hefei Bomei Biotechnology, China). CellTiter-Blue^®^ Cell Viability Assay kit was purchased from Promega for cell viability assay.

### Antibodies and peptides

Actin and Bcl2 were purchased from Sangon Botech (Shanghai, China). Bcl2, PPARα, ubiquitin, and actin were purchased from Santa Cruz. Secondary antibodies were obtained from Jackson Immunoresearch.

### Plasmids

Human PPARα or Bcl2 cDNA was subcloned into pcDNA3 vector. Plasmids were mutated by the site-directed mutagenesis method and were identified by DNA sequencing. Plasmids were transfected by turboFect transfection reagent according to the manufacturer's instructions (Thermo Scientific). PPARα shRNA plasmids (GV248 vector) were purchased from GeneCHEM (China).

### Western blot, immunoprecipitation, subcellular fractionation

Subcellular fractionation extraction or immunoprecipitation was performed as described previously [6]. For denatured immunoprecipitation to disrupt the non-covalent protein interactions, cell extracts were heated at 95°C for 5 min in the presence of 1% SDS, and then tenfold dilution of SDS. The samples were subjected to SDS-PAGE, transferred to a nitrocellulose membrane, then probed by Western blot analysis with the indicated antibody and developed by using an ECL reagent.

### Statistical analysis

Data are expressed as the mean ± SEM. Statistical comparison was carried out with one-way analysis of variance (ANOVA) and Dunnett's test.

## SUPPLEMENTARY FIGURES


