# Supplementary material for: PPARα induces cell apoptosis by destructing Bcl2
Source: Oncotarget. 2015 Nov 9;6(42):44635–42. doi: 10.18632/oncotarget.5988 (PMC4792581; doi:10.18632/oncotarget.5988)
Supplement: Supplementary file 1 [file oncotarget-06-44635-s001.pdf]

SUPPLEMENTARY FIGURES

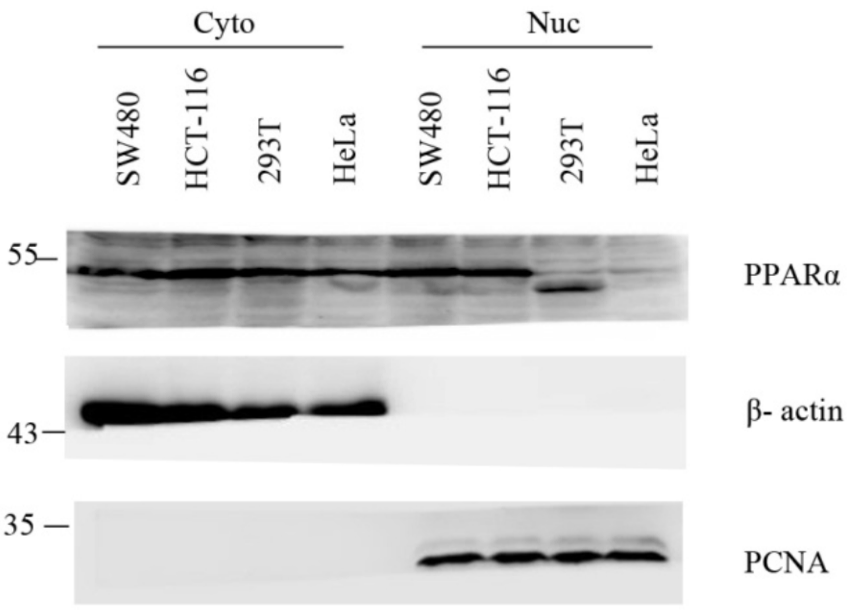

Supplementary Figure S1: Expression of Bcl2 or PPARα in various cancer cell lines. Subcellular extraction of various cancer cells were subjected to Western blot.

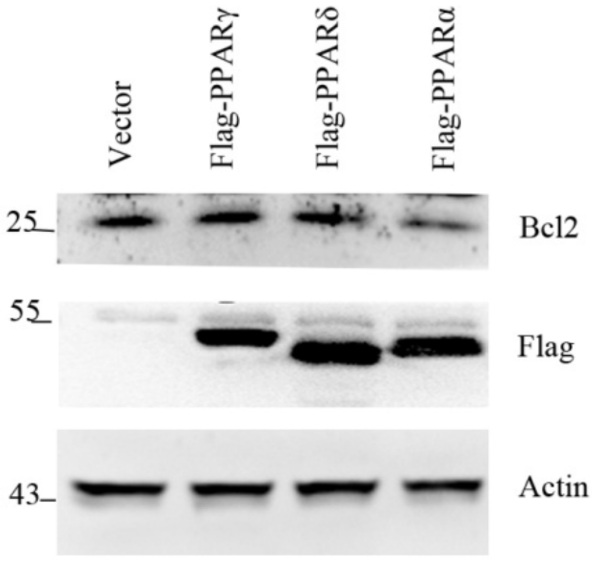

Supplementary Figure S2: PPARα not PPARγ or PPARδ reduces Bcl2 protein levels. HEK293T cells were transfected plasmids as indicated. Cell lysates were subjected to Western blot.

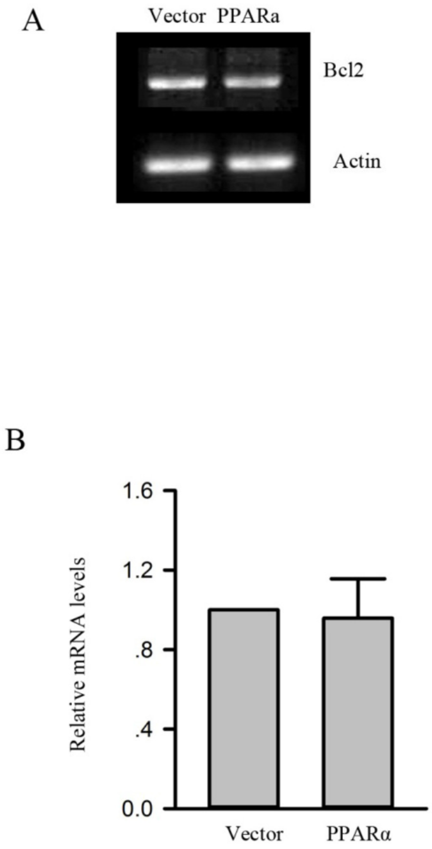

Supplementary Figure S3: PPARα has no effect on Bcl2 gene expression. A, B. SW480 cells were transfected vector or PPARα for 36 h. RT-PCR and real-time PCR were assayed.

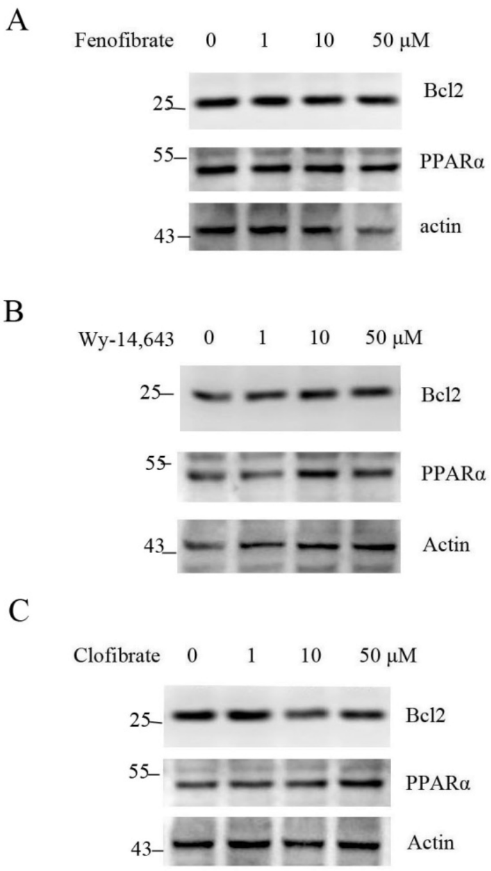

Supplementary Figure S4: PPARα ligands have no effect on Bcl2 protein levels. SW480 cells were treated with fenofibrate, Wy-14,643, or clofibrate as indicated for 1 h. Cell lysates were subjected to Western blot.

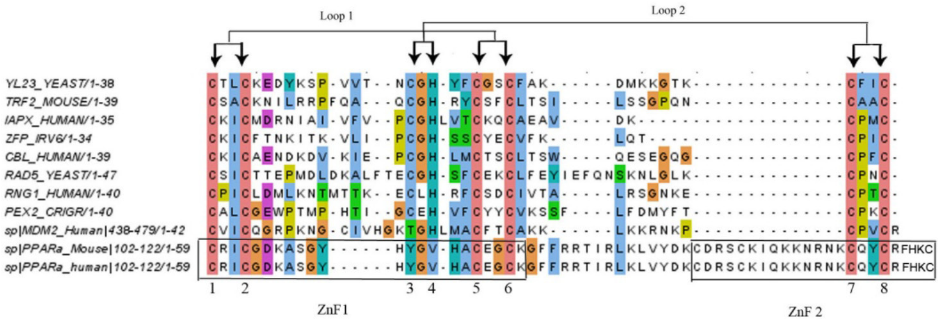

Supplementary Figure S5: PPARα contains RING domains. Alignment analysis was performed among the zinc finger domains in PPARα and the RING domain proteins by using the multiple alignment editor (http://www.jalview.org/download. html).

#	b	Seq.	y
1	114.09	L	
2	227.18	I	1347.70
3	374.24	F	1234.62
4	445.28	A	1087.55
5	502.30	G	1016.51
6	744.44	K*	959.49
7	872.50	Q	717.35
8	985.58	L	589.29
9	1114.63	E	476.21
10	1229.65	D	347.17
11	1286.67	G	232.14
12		R	175.12

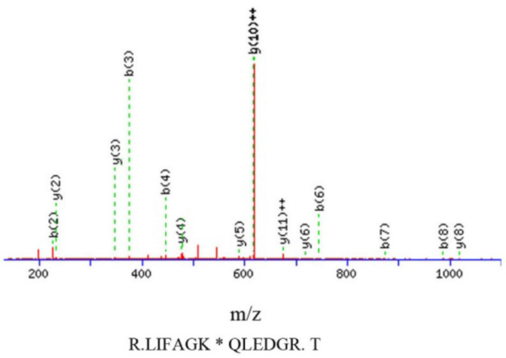

Supplementary Figure S6: PPARα induces K48- linked ubiquitination of Bcl2. SW480 cells were transfected with his-PPARα and cell lysates were subjected to Ni-NTA pull-down and SDS-PAGE. The coomassie stained proteins were excised and subjected to LC/MS/MS analysis. Ubiquitinated peptides are identified by a 114.1 Da diglycine (GG) tag on lysine residues, which is derived from the C-terminus of ubiquitin by trypsin cleavage. The full tryptic peptide LIFAGK* QLEDGR with K48 modified by GG is shown. K* depicts the lysine residue modified by isopeptide linkages.

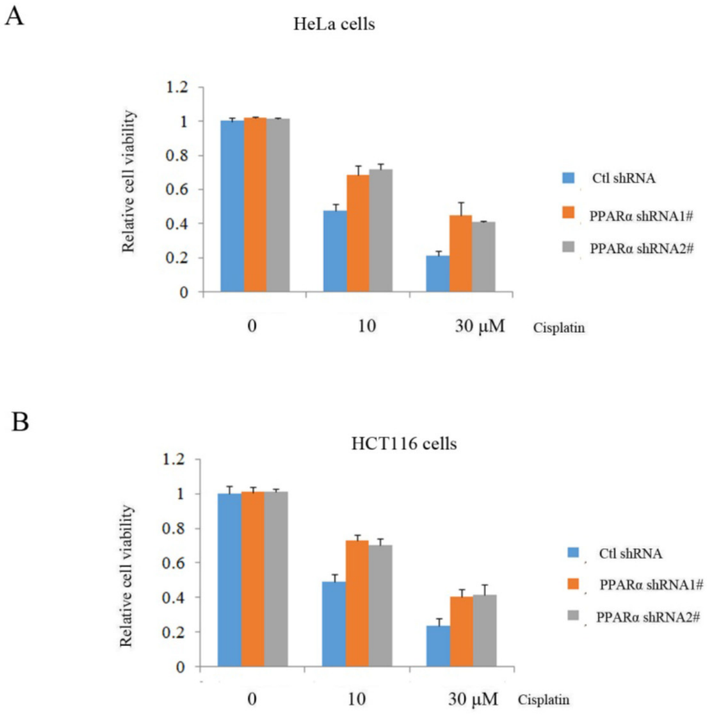

Supplementary Figure S7: PPARα decreases cell survival in response to cisplatinum. PPARα shRNA silenced HeLa **A.** or HCT116 **B.** cells were treated with cisplatinum as indicated for 24 h. Cell viability was assayed (see experimental procedures). Results are expressed as means ± SEM (n = 3).

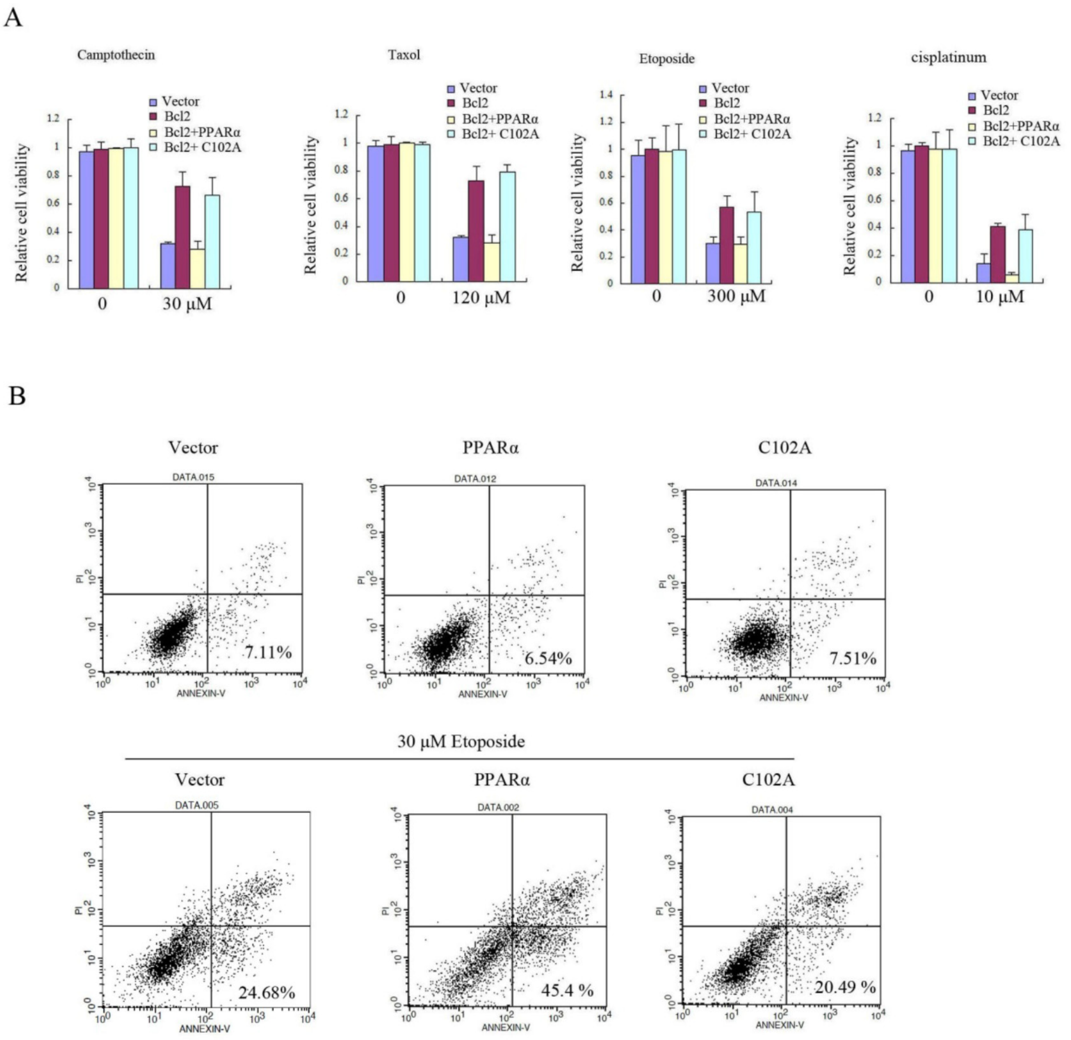

Supplementary Figure S8: C102 is required for PPARα-induced cell apoptosis. A. SW480 cells were transiently transfected with plasmids as indicated. Cells were treated with chemotherapy drugs (cisplatinum, etoposide, taxol, etoposide) for 24 h. Cell viability was assayed (see experimental procedures). Results are expressed as means ± SEM (n = 3). **B.** SW480 cells were transiently transfected with plasmids as indicated. Cells were treated without or with etoposide for 24 h. Cell apoptosis was assayed (see experimental procedures).

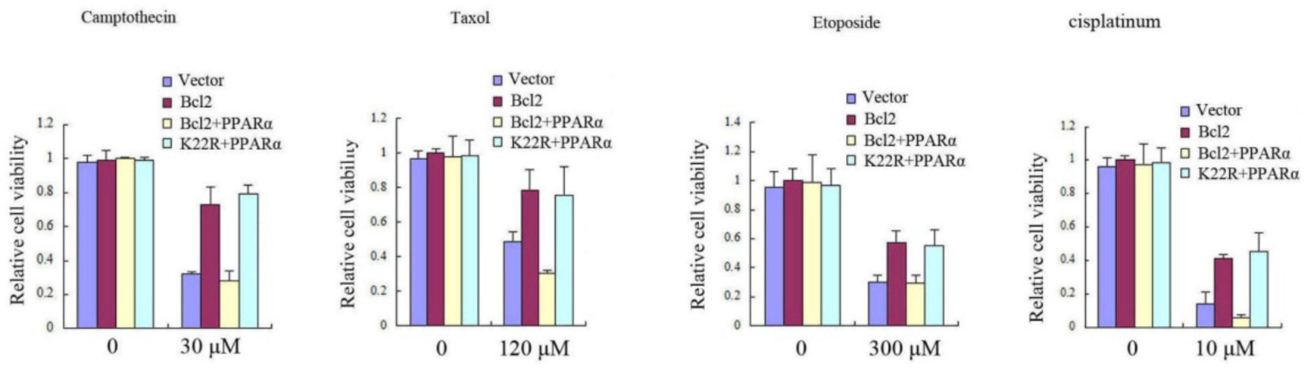

Supplementary Figure S9: PPARα decreases cell survival in response to chemotherapy drugs. SW480 cells were transiently transfected with plasmids as indicated. Cells were treated with chemotherapy drugs (cisplatinum, etoposide, taxol, etoposide) for 24 h. Cell viability was assayed (see experimental procedures). Results are expressed as means ± SEM (n = 3).
